# The Suprachiasmatic Nucleus Regulates Anxiety-Like Behavior in Mice

**DOI:** 10.3389/fnins.2021.765850

**Published:** 2022-01-20

**Authors:** Chelsea A. Vadnie, Kaitlyn A. Petersen, Lauren A. Eberhardt, Mariah A. Hildebrand, Allison J. Cerwensky, Hui Zhang, Jennifer N. Burns, Darius D. Becker-Krail, Lauren M. DePoy, Ryan W. Logan, Colleen A. McClung

**Affiliations:** ^1^Department of Psychology, Ohio Wesleyan University, Delaware, OH, United States; ^2^Translational Neuroscience Program, Department of Psychiatry, Center for Neuroscience, University of Pittsburgh, Pittsburgh, PA, United States; ^3^Department of Pharmacology and Experimental Therapeutics, Boston University School of Medicine, Boston, MA, United States

**Keywords:** suprachiasmatic nucleus, anxiety, mice, optogenetics, circadian rhythms, amplitude

## Abstract

Individuals suffering from mood and anxiety disorders often show significant disturbances in sleep and circadian rhythms. Animal studies indicate that circadian rhythm disruption can cause increased depressive- and anxiety-like behavior, but the underlying mechanisms are unclear. One potential mechanism to explain how circadian rhythms are contributing to mood and anxiety disorders is through dysregulation of the suprachiasmatic nucleus (SCN) of the hypothalamus, known as the “central pacemaker.” To investigate the role of the SCN in regulating depressive- and anxiety-like behavior in mice, we chronically manipulated the neural activity of the SCN using two optogenetic stimulation paradigms. As expected, chronic stimulation of the SCN late in the active phase (circadian time 21, CT21) resulted in a shortened period and dampened amplitude of homecage activity rhythms. We also repeatedly stimulated the SCN at unpredictable times during the active phase of mice when SCN firing rates are normally low. This resulted in dampened, fragmented, and unstable homecage activity rhythms. In both chronic SCN optogenetic stimulation paradigms, dampened homecage activity rhythms (decreased amplitude) were directly correlated with increased measures of anxiety-like behavior. In contrast, we only observed a correlation between behavioral despair and homecage activity amplitude in mice stimulated at CT21. Surprisingly, the change in period of homecage activity rhythms was not directly associated with anxiety- or depressive-like behavior. Finally, to determine if anxiety-like behavior is affected during a single SCN stimulation session, we acutely stimulated the SCN in the active phase (zeitgeber time 14-16, ZT14-16) during behavioral testing. Unexpectedly this also resulted in increased anxiety-like behavior. Taken together, these results indicate that SCN-mediated dampening of rhythms is directly correlated with increased anxiety-like behavior. This work is an important step in understanding how specific SCN neural activity disruptions affect depressive- and anxiety-related behavior.

## Introduction

Circadian rhythms are physiological processes that oscillate with an approximate 24-h period. These rhythms are frequently disrupted in psychiatric disorders, where they are typically dampened or shifted (Souetre et al., 1989; Emens et al., 2009; Hasler et al., 2010; Robillard et al., 2013). For some individuals, environmental circadian disruption, such as shift work and jet lag, can trigger or exacerbate symptoms of psychiatric disorders (Kalmbach et al., 2015; Inder et al., 2016; Lee et al., 2017). Differences in chronotype and disrupted social rhythms are also associated with increased prevalence of depression and/or anxiety (Reid et al., 2012; Fares et al., 2015; Cox and Olatunji, 2019; Mathew et al., 2019; Sabet et al., 2021). Furthermore, treatments that directly affect circadian rhythms, including social rhythm therapy and bright light therapy, have therapeutic effects for some patients (Frank et al., 2005; Penders et al., 2016). These data suggest that circadian rhythm disruption plays a key role in depression and/or anxiety. However, the underlying mechanisms linking rhythm disruption to mood and anxiety disorders remain unclear.

Several human postmortem brain studies have found alterations in specific neurotransmitters and/or receptors in the SCN of the hypothalamus from individuals with a mood disorder (Zhou et al., 2001; Wu et al., 2013, 2017). The SCN acts as the central pacemaker, synchronizing bodily rhythms with each other and the environment. This region is an effective pacemaker because SCN neurons are highly coupled and exhibit a robust circadian rhythm of neuronal firing (Welsh et al., 2010). Specifically, SCN neuronal firing is high during the subjective day (up to 8–12 Hz) and low during the subjective night (typically <1 Hz) in both nocturnal and diurnal species (Inouye and Kawamura, 1979; Sato and Kawamura, 1984; Colwell, 2011). Additionally, the SCN directly receives light information from intrinsically photosensitive retinal ganglion cells (ipRGCs) in the eye that primarily use glutamate as a neurotransmitter (Ebling, 1996; Berson et al., 2002). Glutamatergic signaling in the SCN phase shifts rhythms similar to environmental light (Ding et al., 1994; Shibata et al., 1994; Ebling, 1996). Early in the subjective night, light delays rhythms (signaling the day is longer than expected), whereas late in the subjective night light advances rhythms (signaling the day is starting earlier than expected) (Pittendrigh and Daan, 1976). It is possible that SCN function may be disrupted in individuals with mood and/or anxiety disorders which could affect both how rhythms are entrained to environmental cues and circadian rhythms in brain regions implicated in psychiatric disorders.

At the cellular level, rhythms are generated by transcriptional/translational feedback loops of circadian genes, which are expressed in nearly every cell in the body (Takahashi, 2017). Genetic studies have implicated polymorphisms in several circadian genes in various psychiatric disorders (Soria et al., 2010; Shi et al., 2016; Liberman et al., 2017; Jones et al., 2019). Interestingly, a recent study by Chen and colleagues found that diurnal variations in mood in humans with depression is associated with a polymorphism in a circadian gene, *RORA*, and decreased functional connectivity between the SCN and the superior temporal gyrus (Chen et al., 2021). Thus, this suggests that in some individuals with depression, polymorphisms in circadian genes may disrupt signaling between the SCN and other brain regions. Overall clinical studies suggest that circadian rhythm abnormalities, perhaps through SCN-dependent mechanisms, could be the cause, rather than the effect, of mood or anxiety disorders in some individuals.

Animal studies have shown that altering circadian rhythms can cause increases in anxiety- and/or depressive-like behavior. While changes in rodent anxiety-like avoidance behavior in tests like the elevated plus maze or in rodent depressive-like immobility in the forced swim test are not the same as complex changes in anxiety and mood in humans, these tests have been widely used and have face and predictive validity (Walf and Frye, 2007; Yankelevitch-Yahav et al., 2015). For example, similar to what is thought to occur in humans, environmental circadian disruption, such as repeated advances in the LD schedule, constant light, and non-24 h LD cycles can all disrupt rhythms and increase depressive- and/or anxiety-like behavior in rodents (LeGates et al., 2012; McGowan and Coogan, 2013; Tapia-Osorio et al., 2013; Ben-Hamo et al., 2016; Zhou et al., 2018; Horsey et al., 2019). Furthermore, manipulation of circadian genes can also impact psychiatric-related rodent behavior. Knocking down expression of *Bmal1*, a core circadian gene, in the SCN was shown to increase depressive- and anxiety-like behavior in mice which indicates that molecular rhythms in the SCN play a key role in regulating psychiatric-related behaviors (Landgraf et al., 2016a). Moreover, we have found that disrupting circadian gene expression in brain regions outside of the SCN in mice can also increase depressive- and anxiety-like behavior (Mukherjee et al., 2010; Spencer et al., 2013). One possibility for how this might occur naturally is that environmental disturbances could disrupt SCN rhythms, which in turn alters gene expression rhythms in other parts of the brain, potentially increasing depressive- and anxiety-like behavior. To this point, unpredictable chronic mild stress (UCMS), a well-established paradigm to increase depressive- and anxiety-like behavior, was found to dampen locomotor activity, body temperature, and SCN molecular rhythms in mice (Logan et al., 2015). Interestingly, UCMS also enhanced molecular rhythms in the nucleus accumbens (NAc). Alterations of rhythms both in the NAc and SCN were directly correlated with increases in mouse behavior related to anxiety and depression. Together, these studies suggest that the SCN plays a key role in regulating depressive- and anxiety-like behavior in mice.

In contrast to these findings, recent work shows that irregular light cycles increase depressive-like behavior in mice through an ipRGC-brain pathway independent from the SCN, suggesting that the SCN may not play a major role in regulating light-induced depressive-like behavior (Fernandez et al., 2018). Furthermore, lesioning the SCN generally leads to an antidepressant response in rodents, i.e., less immobility in the forced swim test (FST), suggesting that loss of SCN-mediated synchronization of rhythms would be therapeutic (Arushanyan and Popov, 1995; Tataroglu et al., 2004). One important reason for these conflicting results may be that none of the aforementioned studies directly manipulated the neural activity of the SCN. Since the light and stress manipulations described above can affect the brain in both SCN-dependent and -independent ways, there is a need to understand whether SCN neural activity-mediated rhythm disruption increases depressive- and anxiety-like behaviors.

To determine how SCN-mediated shifting and dampening of rhythms affects psychiatric-related behaviors in mice, we used optogenetics to increase SCN neuronal firing at specific, atypical times of day. We utilized a previously described SCN optogenetic stimulation paradigm (Jones et al., 2015) which was shown to increase SCN neuronal firing and phase shift SCN molecular rhythms similar to what would be expected for the effects of environmental light on the SCN (Jones et al., 2015). Here, our goal was to use a similar SCN optogenetic stimulation paradigm to both acutely and chronically shift or dampen rhythms to determine the specific role of the SCN in regulating depressive- and anxiety-like behaviors. Investigating these mechanisms could lead to the development of new therapeutics for mood and anxiety disorders that directly target the SCN.

## Materials and Methods

### Animals and Housing Conditions

For chronic SCN stimulation, homozygous *Vgat*-Cre mice (B6.FVB.129S6-*Slc32a1^TM 2(cre)Lowl^*/J; The Jackson Laboratory; stock 016962) were crossed with homozygous Cre-dependent ChR2 mice (Ai32, B6.129S-Gt(ROSA)^26*Sortm*32(*CAG–COP*4^*H^134R/EYFP)Hze^/J; The Jackson Laboratory; stock 012569) to yield heterozygous *Vgat*-Cre;ChR2 mice. We used male heterozygous *Vgat*-Cre;ChR2 and homozygous *Vgat*-Cre mice (8–15 weeks old) for implanting optic fibers for the chronic SCN stimulation and the SCN stimulation for c-Fos analyses. To assess the effect of acute SCN stimulation on behavior in the open field (OF), homozygous *Vgat-*Cre mice (B6; stock 028862) were crossed with heterozygous Cre-dependent ChR2 mice (Ai32; stock 024109) to yield heterozygous *Vgat*-Cre;ChR2 B6 mice and heterozygous *Vgat*-Cre B6 littermate controls. These mice were backcrossed onto a C57/BL6J background at The Jackson Laboratory. Male and female heterozygous *Vgat*-Cre;ChR2 B6 and *Vgat*-Cre B6 mice were used between 12–15 weeks old. Mice were maintained on a 12:12 LD schedule (lights on at 7 AM and off at 7 PM, except where described) and were provided with food and water *ad libitum*. All animal use was conducted in accordance with the National Institute of Health guidelines and approved by the Institutional Animal Care and Use Committees of the University of Pittsburgh. Sample sizes were chosen based on adequately powered sizes in our previous studies.

### Surgery

Mice were anesthetized with isoflurane and placed in a stereotaxic device. An optic fiber (NA 0.39, 400 μm core, Thorlabs, Newton, NJ, United States) coupled to a metal ferrule was implanted proximally dorsal to the SCN (AP −0.1 mm, ML 0.0 mm, DV −5.0 mm). Optic fiber implants were adhered to the skull with cement (C&B Metabond, Parkell, Edgewood, NY; black dental cement, Lang Dental Manufacturing, Wheeling, IL, United States). The incision was closed with tissue adhesive (Vetbond, 3M, St. Paul, MN, United States). Light transmission was measured before implantation and postmortem. Only mice with fibers with ≥80% efficiency were used.

### Circadian Activity and Sleep/Wake Recordings

Homecage activity and sleep/wake measurements were determined by piezoelectric recording of movements and breath rate (PiezoSleep, Signal Solutions, LLC, Lexington, KY, United States). Mice that were used for chronic SCN stimulation and acute SCN stimulation for c-Fos analysis were individually housed in four-cage unit polycarbonate boxes in a ventilated, light-controlled cabinet (Tecniplast, Buguggiate, Italy). The light was ∼300 lux during the light phase and ∼0 lux during the dark phase in the cabinets. Mice were randomly assigned to experimental groups to counterbalance cabinet and box position. Each cage rested on a polyvinylidene difluoride (PVDF) square sensor (17.8 × 17.8 cm, 110 μm thick) protected by a thin plastic tray (50.8 μm) (Donohue et al., 2008). A rubber pad between each sensor and the base prevented crosstalk between the cages. The sensors were connected to an amplifier. Pressure signals and breath rates were classified as movements related to activity and inactivity or sleep and wake.

### *In vivo* Light Delivery

A 100 mW 473 nm DPSS laser and a 100 mW 447 nm diode laser (OEM Laser Systems, Midvale, UT, United States) were connected to commutators (Doric Lenses Inc., Quebec, QC, Canada) connected to multi-mode fiber optic black-jacketed patch cords (NA 0.22, 200 μm core, Doric Lenses Inc.). Mice were removed from their home cages using a dim red head lamp. Mice were attached to a patch cord and placed in separate black polycarbonate 20 cm^3^ boxes for chronic stimulation or were directly placed into the OF for acute SCN stimulation during behavioral testing. Heat-shrink tubing blocked blue light leakage from the connection between the patch cord and fiber implant. For chronic SCN stimulation and acute SCN stimulation for c-Fos analysis, mice received 1 h session(s) of blue light pulses (8 Hz, 10 ms pulse width, light intensity at the fiber tip 8–11 mW) or, as a control, were sham stimulated (connected to patch cords, but did not receive blue light pulses). To assess the effect of acute SCN stimulation on anxiety-like behavior in the OF, mice received blue light pulses (8 Hz, 10 ms pulse width, 8–11 mW light intensity at the fiber tip) in alternating 1 min blocks for a total of 6 min during behavior testing. Light spread and power densities (mW/mm^2^) were calculated as described previously (Sidor et al., 2015). Mice that lost fiber implants, had poor fiber placement, or had low fiber efficiency were removed from the study.

### Acute Suprachiasmatic Nucleus Stimulation for c-Fos Analysis

Mice were maintained on a 12:12 LD schedule for 1 week. Mice were then placed in constant darkness (DD) for 5–7 days. Stimulation times were determined by finding three consecutive onsets and using linear regression to predict the onset for the stimulation day (day 6 or day 8). Mice were either stimulated or sham-stimulated at CT21 in the dark using a dim red headlamp. Mice were returned to their homecage and perfused 1 h later under dim red light.

### Immunofluorescence

Mice were deeply anesthetized with pentobarbital (200 mg/kg, *i.p.*) for c-Fos or ketamine (100 mg/kg, *i.p.*) and xylazine (10 mg/kg, *i.p.*) for all other histology. Mice were perfused with ice-cold 1X PBS followed by 4% paraformaldehyde for 10 min. Brains were removed and postfixed overnight in 4% paraformaldehyde. Brains were cryoprotected in 30% sucrose and cut into 40 μm floating coronal sections. Optic fiber placements were assessed relative to ChR2-eYFP in the SCN.

Sections were processed for c-Fos or VGAT labeling to visualize the effect of optogenetic stimulation of the SCN on a marker of neural activity and to verify that ChR2 was expressed in GABAergic neurons, respectively. For these experiments, floating sections were rinsed in 1X PBS and blocked (5% normal donkey serum, 0.2% Triton-X in 1X PBS) for 1 h. Sections were incubated with rabbit anti-c-Fos antibody (1:5000, ABE457, EMD Millipore, Burlington, MA, United States) for 48 h or goat anti-VGAT (D-18) antibody (1:400, sc-49574, Santa Cruz Biotechnology, Dallas, TX, United States) overnight at 4°C on a shaker. Sections were washed in 1X PBS and incubated with donkey anti-rabbit Alexa 555 (1:500, A31572, Invitrogen, Carlsbad, CA, United States) or donkey anti-goat Alexa 555 (1:1000, A21432, Invitrogen) for 2 h at room temperature. Sections were rinsed and mounted on slides.

Slices immunostained for c-Fos were imaged at 10× with a confocal microscope (FV1200 IX83, FluoView, Olympus, Center Valley, PA, United States). Images were obtained using FV10ASW 4.2 software with a 1.5 zoom. A 30 μm z-stack (with 5 μm steps) was obtained for 3–6 sections containing the SCN per sample (5–6 mice/group). ImageJ was used to count the number of c-Fos-positive cells. Counting was carried out by an investigator blinded to the experimental groups. Sections immunostained for VGAT were imaged at 4× magnification on a fluorescent microscope (Olympus).

### Chronic Suprachiasmatic Nucleus Optogenetic Stimulation at CT21

Male *Vgat*-Cre;ChR2 mice were habituated to a reverse LD schedule (lights off at 5 AM and on at 5 PM) for 16 days and were then placed in DD for 5 days to measure baseline free-running rhythms. To shift rhythms, mice received SCN stimulation at circadian time 21 (CT21) every 3 days, where CT12 was defined as activity onset. Based on environmental light studies (Pittendrigh and Daan, 1976; Meijer et al., 1998; Jud et al., 2005), we expected direct optogenetic stimulation of the SCN at CT21 to increase SCN neuronal firing and advance rhythms. For the first stimulation day, stimulation time was determined by finding the onsets of activity on DD days and using linear regression (ClockLab, Actimetrics, Wilmette, IL, United States) to predict the onset for the first stimulation day. For subsequent days, stimulation times were determined by finding the onsets for the 2 days in between stimulations. Behavior testing took place every 3 days during the active phase, following the sixth stimulation as described below. Stimulations continued every 3 days on non-behavior testing days.

To ensure that blue light pulses in the absence of ChR2 were not capable of altering homecage activity rhythms, we implanted optic fibers in a separate group of *Vgat*-Cre;ChR2 heterozygous as well as control *Vgat*-Cre homozygous mice. The experiment was conducted as described above except mice only received six total SCN stimulations. We did not carry out further behavioral testing on the mice in this experiment since there are differences in the genetic backgrounds of *Vgat*-Cre and *Vgat*-Cre;ChR2 mice which we found affected anxiety-like behaviors.

### Chronic Suprachiasmatic Nucleus Optogenetic Stimulation at Unpredictable Times During the Dark Phase

Male *Vgat*-Cre;ChR2 mice were habituated to a reverse LD schedule (lights off at 5 AM and on at 5 PM) for 10 days before baseline rhythms were measured for 7 days. To dampen the amplitude of rhythms, mice received daily SCN optogenetic stimulation at unpredictable times during the dark phase for 8 days. Since SCN neuronal activity is low during the dark phase, stimulations were expected to increase the trough of SCN neuronal activity rhythms and subsequently dampen the amplitude of rhythms. We stimulated at unpredictable times to prevent entrainment to the stimulations. Behavior testing took place on the ninth day during the inactive phase as described below. Daily SCN stimulations continued at unpredictable times during the dark phase throughout behavior testing. The amplitude of activity rhythms was monitored to ensure efficacy of optogenetic stimulations and mice which showed a significant amplitude change moved forward into additional behavioral studies.

### Acute Suprachiasmatic Nucleus Stimulation at ZT14-16

Male and female *Vgat*-Cre;ChR2 B6 (6M, 5F) and *Vgat*-Cre B6 mice (5M, 4F) were individually housed and maintained on a 12:12 LD schedule in standard ventilated cages. Mice were given 10 days to recover from the optic fiber implantation before we assessed the effect of acute SCN stimulation during behavior testing in the OF.

### Behavioral Assays

For stimulations at CT21, behavior testing occurred between CT14-18, during the early active phase. Behavior testing was carried out during the active phase to reduce variability due to differences in test timing and to minimize the effects of the behavior testing on sleep. Mice were given 30 min to habituate to the room and all behavior testing took place under dim red light (<10 lux) to minimize effects of testing on circadian rhythms.

For unpredictable dark phase stimulations, behavior testing took place during the light phase (ZT3-6) to reduce variability due to differences in test timing and to avoid conflict with the daily stimulations during the dark phase. Mice were given 1 h to habituate to the room before testing. Behavior testing for the OF and elevated plus maze (EPM) took place under dim white light (∼20 lux) to promote exploratory behavior. Other behavior tests occurred under standard room lighting.

To determine if acute SCN stimulation during the dark phase affects behavior in the OF, mice received SCN optogenetic stimulation in the OF between ZT14-16. Behavior testing was performed during the early active phase to match the time of testing done for the chronic stimulations at CT21. Mice were given 30 min to habituate to the room and behavior testing took place under dim red light (<10 lux) to minimize effects of the room lighting on the SCN.

#### Locomotor Activity

Mice were placed in clear boxes (field dimensions: 9.5″ × 18.0″) equipped with photobeams to measure horizontal distance traveled for 60 min (Kinder Scientific Smart Cage Rack System, Poway, CA, United States).

#### Open Field

Mice were placed in the corner of a large plastic arena (52 × 52 × 25 cm) with black walls and a clear bottom. The center was a 24 cm × 24 cm square in the middle of the box that was designated with taped gridlines. To determine the effects of the chronic SCN stimulation paradigms on behavior in the OF, mice were allowed to explore the box for 10 min. To determine the effect of acute SCN stimulation during testing, mice were allowed to explore the box for 6 min and received SCN stimulation for three 1 min blocks. Behavior was video recorded and center entries and center time were manually scored by a blinded, trained observer.

#### Elevated Plus Maze

The EPM was elevated 81 cm above the ground and consisted of two open arms perpendicular to two closed arms (arms: 30 × 5 cm). Mice were placed in the center of the maze facing an open arm. Mice explored the maze for 10 min. Behavior was video recorded. Time in the open arms and arm entries were manually scored by a blinded, trained observer.

#### Light/Dark Box

Clear boxes (Kinder Scientific Smart Cage Rack System; field dimensions: 9.5″ × 18.0″) were divided into two equally sized chambers, a black opaque chamber kept in the dark with a lid and a brightly lit chamber (∼880 lux). An opening was present between the two chambers that was equipped with a door. Mice were first placed in the dark chamber for 2 min and then the door was opened between the two sides. Mice were allowed to explore both chambers for 20 min. Photobeams were used to measure the number of entries into and time spent in the light side.

#### Forced Swim Test

Mice were individually placed into glass beakers of water (25–26°C; 18 cm depth) that were separated by dividers to prevent the mice from observing each other during testing. Behavior was video recorded for 6 min. Time spent struggling during the last 4 min of the test and latency to immobility were scored by a blinded, trained observer. Struggling was defined as any movement that was not for the sole purpose of keeping the mouse afloat. Immobility time was calculated by subtracting the time spent struggling from the total time (4 min).

### Statistical Analysis

We quantified homecage activity using WakeActive and ActivityStatistics software, and sleep using SleepStats2p10 software (Signal Solutions, LLC, Lexington, KY, United States). For homecage activity analysis, data were visualized as normalized actograms with 3 min bin sizes in ClockLab (Actimetrics, Wilmette, IL, United States). The period of activity rhythms was determined by applying a least-squares fit to the onset measures. Homecage activity amplitude was quantified using a least-squares fit cosinor analysis similar to cosinor analyses used in previous studies to quantify changes in mouse activity or human actigraphy rhythms (Park et al., 2012; Hopkins et al., 2017; Palmisano et al., 2017; Musiek et al., 2018; Sen et al., 2018). For stimulation at CT21, the period of homecage activity over the analysis range was used for the cosinor fit. For unpredictable dark phase stimulation, a tau of 24 h was used. To obtain additional measures of activity disruption after unpredictable dark phase stimulation, we carried out a non-parametric circadian rhythm analysis in ClockLab to measure relative amplitude, daily fragmentation (intradaily variability) and day-to-day stability (interdaily stability).

The change in homecage activity and sleep measures relative to baseline were determined for each animal. The behavior testing period was not included in the homecage activity and sleep analysis since the mice were removed from the boxes for extended periods of time. Unpaired two-tailed *t*-tests were used to assess differences in measures between the control and stimulated *Vgat*-Cre;ChR2 mice. Paired two-tailed *t*-tests were used to assess differences in behavior of *Vgat*-Cre B6 and *Vgat*-Cre;ChR2 mice in the OF during acute SCN stimulation blocks relative to the no stimulation blocks. Where there was unequal variance, Welch’s *t*-tests were used and where there was not a normal distribution, Mann-Whitney tests were used. Two-way ANOVAs followed by Tukey *post hoc* tests where appropriate were used to determine differences in homecage activity measures for the control chronic stimulation experiment at CT21 using *Vgat*-Cre and *Vgat*-Cre;ChR2 mice. Correlations between homecage activity measures and behavior were assessed using two-tailed Pearson correlations. Data were analyzed using Prism 7 for PC (GraphPad Software, San Diego, CA, United States). Bar graphs are presented as mean ± SEM and *p* < 0.05 was considered significant.

## Results

### Chronic Suprachiasmatic Nucleus Optogenetic Stimulation at CT21 Shortens the Period and Dampens the Amplitude of Homecage Activity Rhythms

To selectively stimulate neurons in the SCN, we generated heterozygous *Vgat*-Cre;ChR2 mice. VGAT is a vesicular transporter that loads GABA and glycine into synaptic vesicles. Since nearly all SCN neurons are GABAergic (Belenky et al., 2008), this was an effective approach to obtain high ChR2 expression in the SCN (Supplementary Figure 1A). Optic fibers were implanted proximally dorsal to the SCN. High expression of ChR2 in the SCN coupled with limited spread of blue light in the brain allowed us to specifically target the SCN (Supplementary Figure 1B).

**FIGURE 1:**
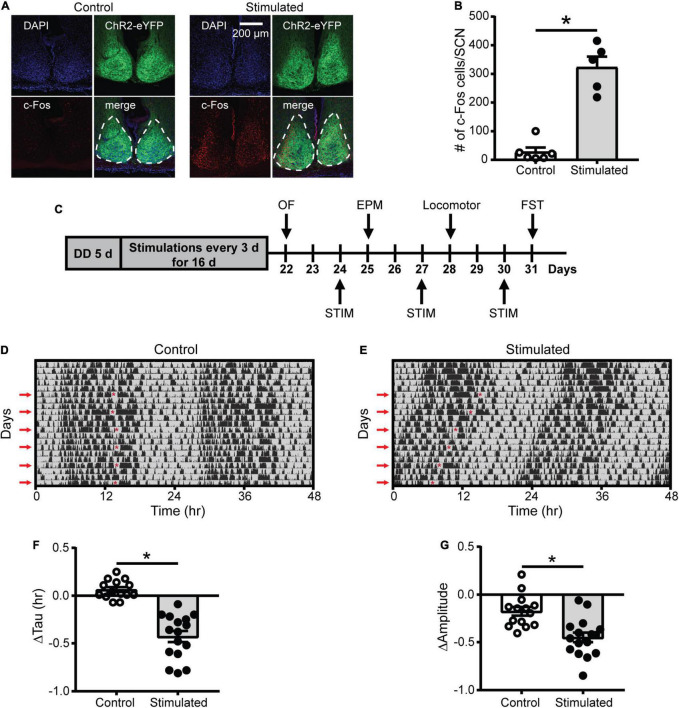
Effects of SCN stimulation at CT21 on c-Fos cells in the SCN and homecage activity rhythms. **(A)** Representative confocal images of coronal mouse brain slices containing the SCN from a control and stimulated mouse. **(B)** The average number of c-Fos-positive cells per mouse SCN were increased after stimulation at CT21. *n* = 5–6 mice. **(C)** Experimental design to determine the effects of repeated SCN stimulation at CT21 on psychiatric-related behaviors. STIM, stimulation. **(D,E)** Representative double-plotted actograms of homecage activity of a control and stimulated mouse. Gray shading indicates when lights were off and red arrows indicate the days when stimulations or sham stimulations occurred at CT21. The red asterisks on the left sides of the actograms indicate the times of the stimulations. Change in homecage activity circadian parameters were measured relative to baseline in DD. **(F)** Stimulated mice exhibited a greater reduction in homecage activity period or tau. **(G)** Stimulated mice showed a greater decrease in homecage activity amplitude. *n* = 14–16 *Vgat*-Cre;ChR2 mice. *p < 0.05.

To determine whether optogenetic stimulation of the SCN increases SCN neuronal activity, we quantified the number of c-Fos-positive cells (a marker of neuronal activity) in the SCN of *Vgat*-Cre;ChR2 mice 1 h after stimulation or sham stimulation at CT21 ended. Consistent with previous work showing that a similar SCN optogenetic paradigm increased SCN neuronal firing (Jones et al., 2015), SCN stimulation at CT21 (late in the active phase of mice when SCN neuronal firing is low) increased the number of c-Fos-positive cells in the SCN relative to controls (Figures 1A,B, Supplementary Table 1, and Supplementary Figure 1C).

Since light at CT21 is known to advance rhythms, which is largely mediated by increased glutamate release in the SCN (Pittendrigh and Daan, 1976; Ding et al., 1994; Shibata et al., 1994; Ebling, 1996), we wanted to determine if repeated optogenetic stimulation of the SCN at CT21 would result in advancing homecage activity rhythms (Figure 1C and Supplementary Figures 1D–F). Stimulation at CT21 did shorten the homecage activity period or tau relative to controls (Figures 1D–F). Consistent with studies showing that shifts in the LD schedule can dampen the amplitude of rhythms (Filipski et al., 2004; Logan et al., 2012), SCN stimulation at CT21 also dampened the amplitude of homecage activity rhythms relative to controls (Figure 1G). The slight reduction in amplitude of the control mice relative to baseline (−0.176 ± 0.046 a.u.) was likely due to interruption in homecage recording for sham stimulation and handling stress. We did not observe a significant change in the average daily percent time mice spent sleeping (Supplementary Figure 2A). However, there was a reduction in average daily sleep bout duration relative to controls (Supplementary Figure 2B). Together our data suggest that chronic SCN stimulation at CT21 altered the pattern of homecage activity with no effect on total sleep time.

As an additional control, we stimulated or sham stimulated *Vgat*-Cre homozygous or *Vgat*-Cre;ChR2 heterozygous mice to ensure blue light pulses in the absence of ChR2 were not affecting homecage activity rhythms. Only mice that expressed ChR2 and received blue light pulses showed a significant shortening and dampening of homecage activity rhythms, confirming that we were not observing effects of blue light pulses alone on rhythms (Supplementary Figures 3, 4).

### Dampened Amplitude of Homecage Activity Rhythms After Suprachiasmatic Nucleus Stimulation at CT21 Was Associated With Increased Anxiety-Like Behavior in the Open Field

**FIGURE 2:**
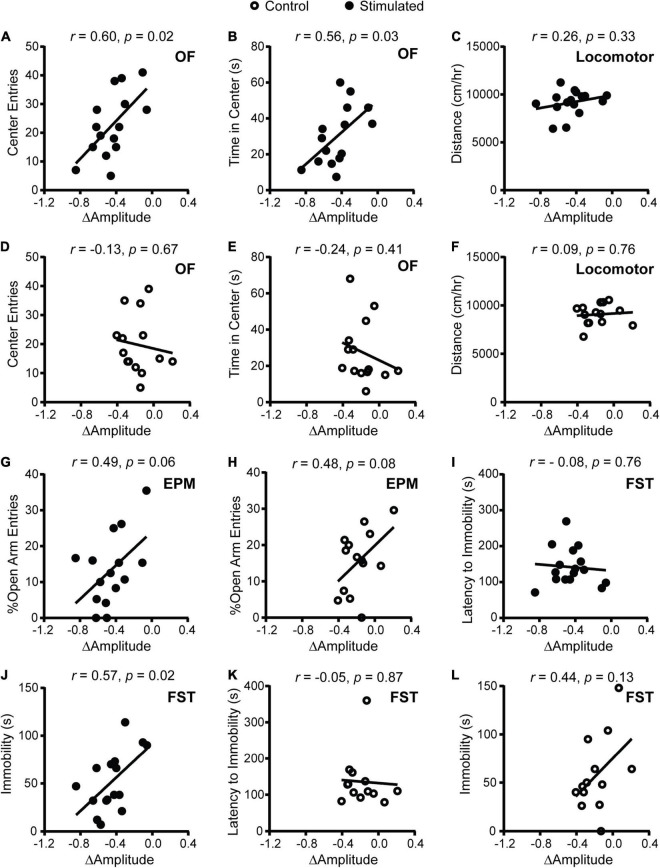
Correlations between change in homecage activity amplitude and behavior in mice that received stimulations or sham stimulations of the SCN at CT21. **(A,B,D,E)** OF, **(C,F)** locomotor, **(G,H)** EPM, **(I–L)** FST. A dampened amplitude of homecage activity rhythms in stimulated mice was correlated with increased anxiety-like behavior in the OF and decreased immobility in the FST. *n* = 13–16 *Vgat*-Cre;ChR2 mice.

To determine whether SCN-mediated disruption of rhythms causes alterations in psychiatric-related behaviors, we examined correlations between homecage circadian activity measures and behavior. In stimulated *Vgat*-Cre;ChR2 mice only, dampened homecage activity rhythms relative to baseline were correlated with fewer entries and less time in the center of the OF (Figures 2A,B). The stimulated *Vgat*-Cre;ChR2 mouse that had the greatest reduction in home cage activity amplitude (−0.848 a.u.) made seven entries into the center of the OF and spent 11.29 s in the center of the OF. In comparison, the stimulated *Vgat*-Cre;ChR2 mice with the smallest reductions in homecage activity amplitude (−0.059 and −0.105 a.u.) made 28 and 41 entries into the center of the OF and spent 37 and 46 s in the center of the OF, respectively. Distance traveled in a novel environment was not correlated with change in homecage activity amplitude (Figure 2C), indicating that correlations with OF behavior were independent from any effect of SCN stimulation on general locomotion. In sham-stimulated *Vgat*-Cre;ChR2 control mice, there were no correlations between change in homecage activity amplitude and behavior in the OF or locomotion in a novel environment (Figures 2D–F). In stimulated mice, there was a strong trend for more dampened homecage activity rhythms to correlate with fewer percentages of open arm entries (Figure 2G). However, a similar trend was observed in controls (Figure 2H) and no correlations were observed with open arm time (data not shown). Surprisingly, we did not observe correlations between changes in the period of homecage activity rhythms and behavior (Supplementary Figure 5). Taken together, our data indicate that dampened homecage activity rhythms due to chronic SCN stimulation at CT21 are associated with increased anxiety-like behavior in the OF.

In stimulated mice (Figures 2I,J), dampened homecage activity amplitude also correlated with reduced immobility in the FST but not latency to immobility. In control mice, change in homecage activity amplitude was not correlated with behavior in the FST (Figures 2K,L).

### Unpredictable Stimulation of the Suprachiasmatic Nucleus During the Dark Phase Dampened the Amplitude of Homecage Activity Rhythms

**FIGURE 3:**
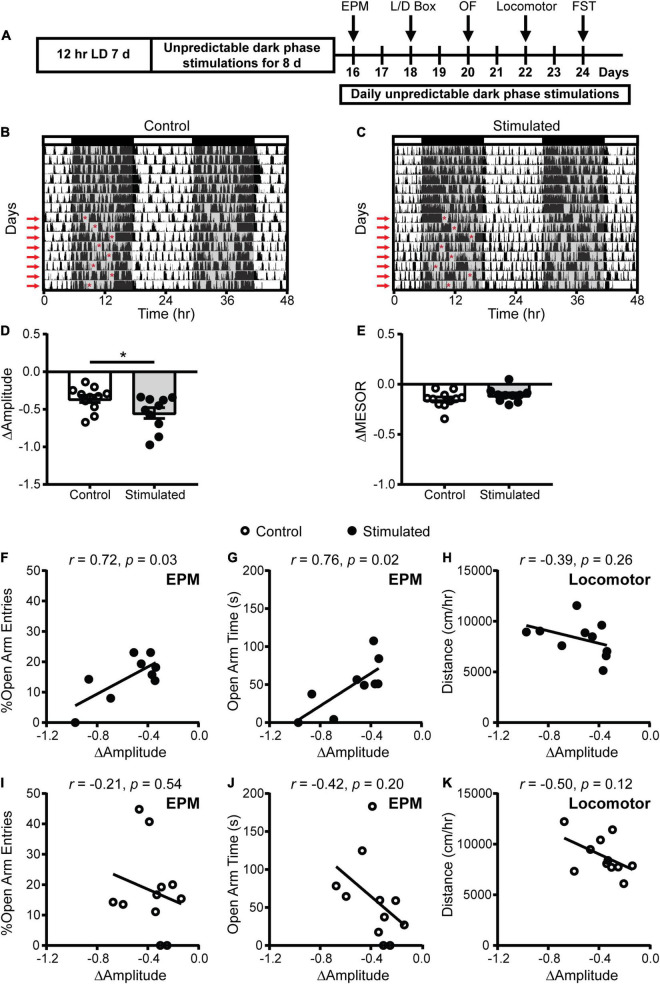
Homecage circadian activity changes and correlations with behavior in mice that received stimulations or sham stimulations of the SCN at unpredictable times during the dark phase. **(A)** Experimental design. **(B,C)** Representative double-plotted actograms of homecage activity of a control and stimulated mouse. Gray shading indicates when lights were off and red arrows indicate when the daily stimulations took place. The red asterisks on the left sides of the actograms indicate the times of the stimulations. Change in homecage activity circadian parameters were measured relative to baseline in LD. **(D)** Stimulated mice exhibited a greater reduction in homecage activity amplitude. **(E)** There was no difference in the change in midline estimating statistic of rhythm (MESOR) between control and stimulated mice. Correlations between change in homecage activity amplitude and **(F,G,I,J)** EPM and **(H,K)** locomotor behavior in mice that received daily unpredictable dark phase stimulations or sham stimulations of the SCN. *n* = 9–11 *Vgat-Cre;ChR2 mice*. *p < 0.05.

To more specifically determine the effects of SCN-mediated disruption of the amplitude of rhythms on mood and anxiety-related behaviors, we kept mice on a 12:12 LD schedule and stimulated the SCN daily at unpredictable times during the dark phase when SCN neuronal firing is normally low (Figure 3A). As expected, stimulated mice exhibited a significantly greater decrease in homecage activity amplitude relative to controls (Figures 3B–D). The decrease in homecage activity amplitude in stimulated mice cannot be attributed to an overall decrease in homecage movement since we did not find a significant difference in midline estimating statistic of rhythm (MESOR) between the groups (Figure 3E). In addition, there were no differences in the change in average daily percent sleep (Supplementary Figure 6A) or average daily sleep bout duration (Supplementary Figure 6B), indicating that the reduction in homecage activity amplitude in stimulated mice is not due to a change in total sleep.

### Dampened Amplitude of Homecage Activity Rhythms With Unpredictable Dark Phase Stimulation of the Suprachiasmatic Nucleus Was Associated With Increased Anxiety-Like Behavior in the Elevated Plus Maze

**FIGURE 4:**
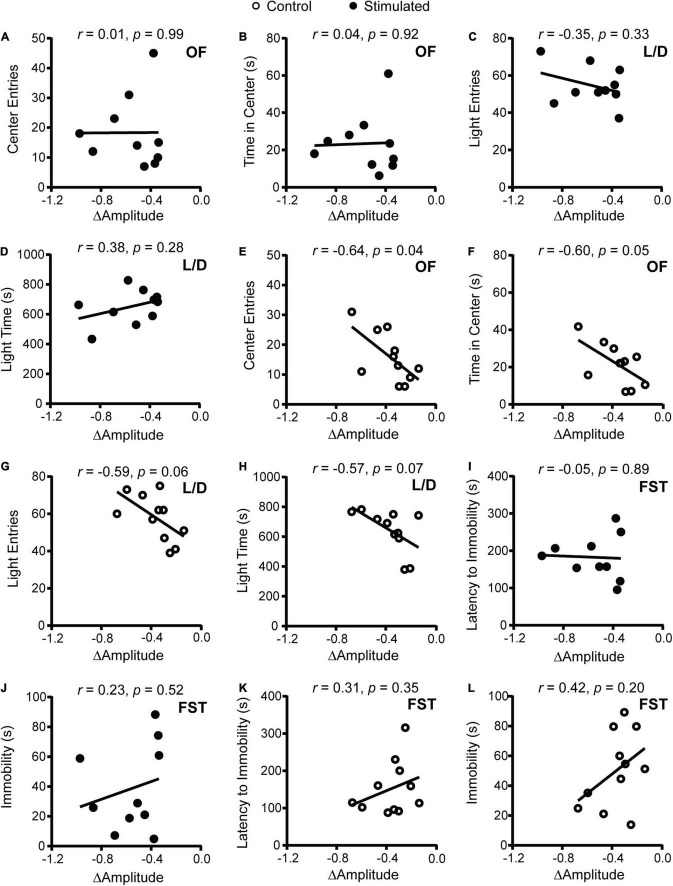
Correlations between change in homecage activity amplitude and behavior in mice that received stimulations or sham stimulations of the SCN at unpredictable times during the dark phase. **(A,B,E,F)** OF, **(C,D,G,H)** LD box, and **(I–L)** FST behavior. A dampened amplitude of homecage activity rhythms in control mice was associated with decreased anxiety-like behavior in the OF. *n* = 9–11 *Vgat*-Cre;ChR2 mice.

To determine how SCN-mediated dampening of rhythms affects anxiety- and depressive-like behaviors, we performed correlations between the change in homecage activity amplitude and behavior. In stimulated mice, dampened homecage activity rhythms were strongly correlated with reduced open arm entries and open arm time in the EPM (Figures 3F,G and Supplementary Figure 6C). The stimulated *Vgat*-Cre;ChR2 mouse that had the greatest reduction in home cage activity amplitude (−0.973 a.u.) did not enter the open arms of the EPM. In comparison, the stimulated *Vgat*-Cre;ChR2 mice with the smallest reductions in homecage activity amplitude (−0.337 and −0.343 a.u.), had open arm entries of 18.18% and 13.79% with open arm times of 84.18 s and 51.12 s, respectively. Distance traveled in a novel environment was not correlated with the change in homecage activity amplitude in stimulated mice (Figure 3H), indicating that the observed correlations were independent from any effect of SCN stimulation on general locomotion. In control mice, no correlations were observed between changes in homecage activity amplitude and behavior in the EPM or distance traveled in a novel environment (Figures 3I–K). Surprisingly, in stimulated mice we did not observe correlations between homecage activity amplitude and other measures of anxiety-like behavior (Figures 4A–D). However, in control mice, dampened amplitude of homecage activity rhythms was correlated with increased center entries and center time in the OF (Figures 4E,F). There were also trends for correlations between dampened amplitude of homecage circadian rhythms and behavior in the LD box in control mice (Figures 4G,H). Thus, in sham-stimulated mice, handling-induced dampening of homecage activity rhythms was associated with less anxiety-like behavior. In both stimulated and control mice, there were no correlations between change in the amplitude of homecage activity rhythms and behavior in the FST (Figures 4I–L). Together our data suggest that dampening the amplitude of rhythms by unpredictable dark phase stimulation of the SCN increases anxiety-like behavior most strongly in the EPM with no impact on depression-like behavior in the FST.

**FIGURE 5:**
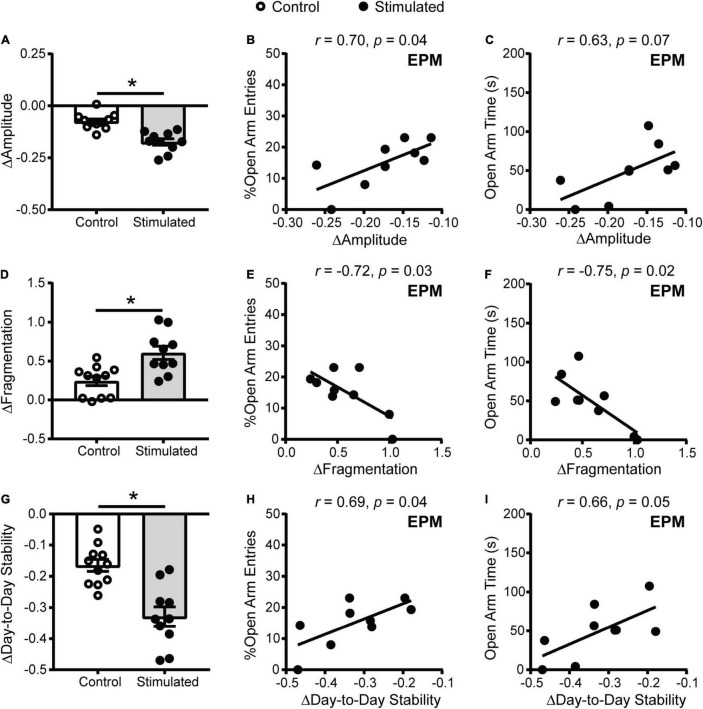
Homecage activity non-parametric circadian parameter changes and correlations with behavior in mice that received stimulations of the SCN at unpredictable times during the dark phase. Change in homecage activity non-parametric circadian parameters were measured relative to baseline in LD. **(A)** Stimulated mice had a greater reduction in homecage activity amplitude, **(D)** increase in fragmentation and **(G)** decrease in day-to-day stability. Correlations between the change in **(B,C)** homecage activity amplitude, **(E,F)** fragmentation and **(H,I)** day-to-day stability with behavior in the EPM in stimulated mice. *n* = 9–11 *Vgat*-Cre;ChR2 mice. *p < 0.05.

One explanation for why homecage activity rhythms are dampened with daily unpredictable dark phase stimulation of the SCN is that our stimulation paradigm disrupts the day/night pattern of rhythms, increasing daily rhythm fragmentation. In addition, we expected that direct stimulation of the SCN during the dark phase would shift rhythms day-to-day (reduce day-to-day stability). To assess daily rhythm fragmentation and day-to-day stability we used a non-parametric circadian rhythm analysis that is frequently used to quantify changes in human actigraphy data. Similar to the cosinor analysis (Figure 3D), the non-parametric analysis revealed that homecage activity amplitude was further decreased in stimulated mice relative to controls (Figure 5A). Consistent with the cosinor analysis (Figures 3F,G), dampened non-parametric activity amplitude was correlated with reduced open arm entries and there was a trend for a correlation with open arm time in the EPM in stimulated mice (Figures 5B,C). Stimulated mice also had a greater increase in homecage activity rhythm fragmentation relative to controls (Figure 5D) which was correlated with reduced open arm entries and open arm time in the EPM (Figures 5E,F). Lastly, stimulated mice had a greater decrease in day-to-day stability of activity relative to controls (Figure 5G). Decreased homecage activity day-to-day stability was also correlated with reduced open arm entries and there was a trend for a correlation with open arm time in the EPM in stimulated mice (Figures 5H,I). We did not see correlations between the non-parametric circadian rhythm measures and locomotion in a novel environment in stimulated mice or behavior in the EPM in controls (Supplementary Figure 7).

### Acute Suprachiasmatic Nucleus Stimulation Increases Anxiety-Like Behavior in the Open Field

**FIGURE 6:**
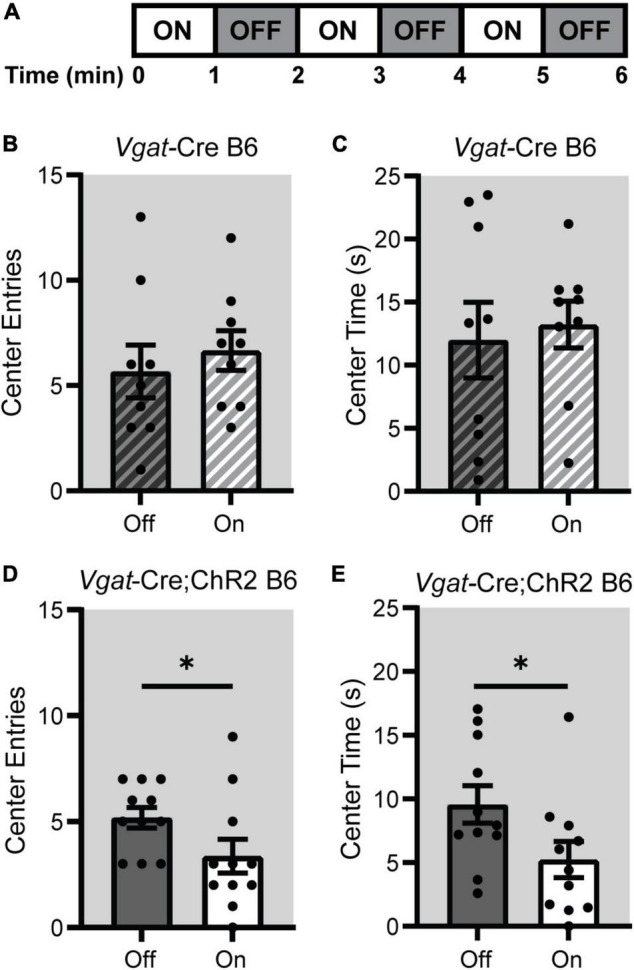
Acute optogenetic stimulation of the SCN during the dark phase increased anxiety-like behavior in the OF. **(A)** Male and female *Vgat*-Cre;ChR2 B6 or *Vgat*-Cre B6 control mice received blue light pulses (8 Hz, 10 ms pulse width) in alternating 1 min blocks for a total of 3 min while in the OF. **(B)** Acute SCN stimulation at ZT14-16 had no effect on center entries or **(C)** total time spent in the center of *Vgat*-Cre B6 mice in the OF. **(D)** In *Vgat*-Cre;ChR2 B6 mice, acute SCN stimulation at ZT14-16 decreased the number of center entries in the OF and **(E)** decreased total time spent in the center time relative to the laser off periods. *n* = 9–11. *p < 0.05.

We next wanted to determine if an acute SCN stimulation during behavioral testing would produce any effects on anxiety-like behavior. To do this we stimulated the SCN of male and female *Vgat*-Cre B6 and *Vgat*-Cre;ChR2 B6 mice for three 1 min blocks while they were exploring the OF for 6 min during the early part of the dark phase, ZT14-16 (Figure 6A). We chose to look at behavior in the OF since this test was easier to carry out with mice tethered to the laser during testing. As expected, stimulating the SCN of *Vgat*-Cre B6 controls had no effect on the number of center entries or time in the center of the OF (Figures 6B,C). Interestingly however, *Vgat*-Cre;ChR2 mice showed fewer center entries and less time spent in the center when the SCN was being stimulated, suggesting that anxiety-like behavior is increased acutely during SCN stimulation (Figures 6D,E).

## Discussion

In this study, we used optogenetics to investigate how SCN-mediated chronic advancing and dampening of rhythms affects psychiatric-related behaviors in mice. Of note, this is one of only a handful of studies that have employed optogenetics in the SCN. Specifically, stimulation of the SCN at CT21 shortened the period and dampened the amplitude of homecage activity rhythms. Stimulation of the SCN at unpredictable times during the dark phase dampened the amplitude, increased the daily fragmentation, and reduced the day-to-day stability of homecage activity rhythms. Neither paradigm significantly altered average daily sleep time, suggesting our SCN stimulation paradigms were primarily affecting homecage activity patterns and not total sleep. In both SCN chronic stimulation paradigms, dampened amplitude of homecage activity rhythms were associated with increased anxiety-like behavior. Furthermore, acute SCN stimulation at ZT14-16 during OF behavioral testing also led to increased anxiety-like behavior, suggesting that SCN stimulation-induced dampening of rhythms directly increases anxiety-related behavior.

In humans, studies show that circadian rhythms are disrupted in psychiatric disorders (Vadnie and McClung, 2017; Logan and McClung, 2019), but few studies have examined anxiety specifically. Some studies suggest that environmental circadian disruption, such as by shift work, increases anxiety (Selvi et al., 2010; Kalmbach et al., 2015; Booker et al., 2019). Genetic circadian disturbances, specifically variants in the circadian genes *PER3* and *ARNTL2*, have been linked with anxiety (Sipila et al., 2010; Liberman et al., 2017). Differences in chronotype and disrupted social rhythms are also associated with increased prevalence of anxiety disorders (Reid et al., 2012; Fares et al., 2015; Cox and Olatunji, 2019; Mathew et al., 2019; Sabet et al., 2021). Finally, consistent with our findings in mice, more dampened or fragmented actigraphy rhythms have been found in individuals with anxiety disorders (Luik et al., 2015; Difrancesco et al., 2019). Thus, although there are a limited number of studies on anxiety specifically, work thus far suggests that circadian rhythm disruptions may play a causal role in anxiety disorders in humans.

In animals, both genetic and environmental disruption of circadian rhythms can increase anxiety-like behavior, but the mechanism is unclear (De Bundel et al., 2013; McGowan and Coogan, 2013; Tapia-Osorio et al., 2013; Qiu et al., 2019). Our findings suggest that SCN-driven dampening of the amplitude of rhythms increases anxiety-like behavior. Consistent with our findings, we previously found that in stressed mice, more dampened SCN molecular rhythms were directly associated with increased anxiety-like behavior, suggesting a role for the amplitude of SCN rhythms in regulating anxiety (Logan et al., 2015). To determine if disrupting SCN molecular rhythms can cause behavioral changes, Landgraf and colleagues knocked down the core circadian gene *Bmal1* in the SCN and found that it dampened the amplitude of a bioluminescent marker of SCN molecular rhythms and increased anxiety-like behavior in mice (Landgraf et al., 2016a). Together, our work, along with previous studies, support the hypothesis that SCN-mediated dampening of rhythms increases anxiety-like behavior in rodents. In future studies, we will determine how SCN optogenetic stimulation affects SCN circadian gene expression and if SCN gene expression changes are associated with other psychiatric-related behaviors in mice.

Previous studies showed that exposing animals to an advancing LD schedule for weeks increased depressive and/or anxiety-like behavior which is why we chose to determine the effects of an advancing SCN stimulation paradigm (McGowan and Coogan, 2013; Horsey et al., 2019). Unexpectedly, we did not observe correlations between changes in period and psychiatric-related behaviors in the mice that received SCN stimulation at CT21. We may have seen associations between period and behavioral measures with a longer stimulation paradigm. However, lengthening our stimulation paradigm is extremely challenging since the animals are free-running and stimulated by the experimenter at the appropriate circadian time of each mouse. As basic research technology progresses, in future studies, a closed-loop wireless SCN optogenetic stimulation approach would allow for longer duration paradigms. It is also possible that a longer, but not a shorter period is associated with anxiety- and depressive-like behavior. In humans, an evening chronotype is more consistently associated with adverse mental health outcomes (Fares et al., 2015; Cox and Olatunji, 2019) and thus we are also interested in determining how SCN-mediated delaying of rhythms would affect behavior.

It is interesting that dampened amplitude of rhythms after repeated SCN stimulation was not correlated with increases in all anxiety-like measures and the specific anxiety-like measures were not consistent across both stimulation paradigms. Although the EPM, OF, and LD box tests are all approach/avoidance behavior procedures, there is evidence to support that each individual test measures distinct aspects of anxiety-like behavior. Furthermore, anxiety-like measures from these approach/avoidance tests are often unrelated (Ramos et al., 1998; Lalonde and Strazielle, 2008). Thus, these approach/avoidance behaviors may differentially assess types of anxiety-like behavior (i.e., fear of open, brightly lit, or elevated spaces) and the two chronic SCN optogenetic stimulation paradigms used here may have differentially affected these measures. An important point is that the designs of the two experiments were very different (e.g., stimulation every 3 days versus daily, housed in DD versus LD, timing of the behavior testing, etc.) and thus it is not entirely unexpected that we did not observe the same correlations across the two experiments. In addition, the combined effect of shortening the period and dampening the amplitude in one experiment versus primarily dampening the amplitude in the other may have led to distinct neuronal outcomes which manifested in increasing a particular anxiety-related measure. In future studies, it will be interesting to try to tease apart how period and amplitude changes together might impact other behaviors compared to a reduction of amplitude or change in period alone.

Interestingly, unpredictable SCN sham stimulations resulted in correlations between dampened amplitude of homecage activity rhythms with less anxiety-like behavior in the OF. An important point is that the amplitude measure is the change in homecage activity amplitude relative to baseline. A small reduction in amplitude is expected in control animals due to handling stress and removal from PiezoSleep boxes for sham stimulation. It is known that repeated handling can reduce anxiety-like behavior (Schmitt and Hiemke, 1998; Costa et al., 2012). In control mice, having slightly less robust, more flexible rhythms under times of acute stress may be beneficial. There is evidence to support that having a smaller amplitude of SCN rhythms, intercellular desynchrony of the SCN, leads to a more flexible SCN that can adapt to environmental changes (Noguchi et al., 2018). Another possibility is that an SCN-independent mechanism underlies the relationship between the change in amplitude in homecage activity rhythms and anxiety-like behavior in control animals. Acute handling stress through an SCN-independent mechanism may both reduce anxiety-like behavior and the amplitude of homecage activity rhythms. Stress typically dampens homecage activity rhythms, but the effects on SCN rhythms seem to depend on the type and duration of stress (Jiang et al., 2011; Tahara et al., 2015; Landgraf et al., 2016b). In our hands, more frequent and prolonged stress dampened rhythms in the SCN and increased anxiety-like behavior (Logan et al., 2015). Thus, there may be an inverse U-shape relationship between homecage activity amplitude and anxiety-like behavior. Having a decreased amplitude of homecage activity rhythms (perhaps through an SCN-independent mechanism) may be beneficial to allow for more flexibility under acute stress which would lead to less anxiety-like behavior. However, having a significant SCN-driven dampening of rhythms may increase anxiety-like behavior perhaps through disrupting activity in many downstream brain regions.

For the FST, we only observed a correlation between homecage activity amplitude and a traditional measure of behavioral despair in mice stimulated at CT21. We did not observe correlations between change in homecage activity amplitude and behavior in the FST with stimulation at unpredictable times during the dark phase. Contrary to what we hypothesized, with SCN stimulation at CT21, more dampened homecage activity rhythms were associated with less immobility in the FST, less behavioral despair. Interestingly, a recent study similarly described that light exposure in the late part of the dark phase (ZT22) induces less immobility in the FST in mice. In this same study, loss of the circadian gene *Per1* was shown to increase depressive- and anxiety-like behavior in mice. Intriguingly, *Per1* in the lateral habenula was necessary for the antidepressant effect of light but loss of *Per1* in the lateral habenula did not affect anxiety-like behavior in the O-maze, indicating that increased anxiety-like behavior observed in *Per1* global knockout mice is attributed to loss of *Per1* somewhere else in the brain, potentially the SCN (Olejniczak et al., 2021). Thus, SCN-mediated dampening of rhythms may not play a strong role in regulating depressive-like behavior. In fact, Fernandez and colleagues showed that mice exposed to an ultradian LD cycle display increased behavioral despair only when ipRGCs that project to brain regions outside of the SCN were left intact, indicating that the SCN does not play a major role in regulating depressive-like behavior induced by light (Fernandez et al., 2018). Rather the SCN may play a greater role in regulating anxiety-like behavior.

In support of this theory, our acute SCN optogenetic experiment revealed that SCN stimulation during the early part of the dark phase (ZT14-16) is sufficient to increase anxiety-like behavior in the OF. This is, to our knowledge, the first study to acutely activate the SCN and perform behavioral testing for anxiety-related behavior. In the future, it will be necessary to determine the effects of acute SCN stimulation during behavioral testing at other time points to fully understand how SCN disruption could be leading to alterations in anxiety-related behaviors. Furthermore, potential impacts of SCN stimulation on other regions of the hypothalamus, such as the PVN, and traditional “anxiety-related” regions such as the amygdala will be explored. Our acute results suggest that in our chronic SCN stimulation paradigms, the mice that experienced the greatest SCN-mediated disruption to their circadian rhythms may be displaying a lasting increase in their anxiety-like behavior since behavior testing took place outside of the SCN stimulation sessions. We are interested in determining how long after chronic SCN stimulation cessation do anxiety-like behaviors persist.

We recognize that a limitation of this study is that the experiments involved assessing correlations with behavioral measurements in short assays that are not the same as humans experiencing long-lasting changes in mood and anxiety. We chose these behavior assays because they are classic, widely used assays of anxiety- and depressive-like behavior in rodents. However, it has been brought to the forefront that there are weaknesses associated with the interpretation and reliability of these tests, especially the FST (Anyan and Amir, 2018). Thus, in future studies it will be necessary to assess other acute and chronic changes in depressive- and anxiety-like measures (e.g., sucrose preference, learned helplessness, comprehensive homecage phenotyping) in mice after manipulation of SCN neuronal activity.

Our findings ultimately suggest that SCN-mediated dampening of rhythms can increase measures of anxiety-like behavior in mice. This work is an important step into understanding how the SCN regulates psychiatric-related behaviors. The SCN consists of a diverse population of neurons that receive different inputs, express different neuropeptides, and have distinct neural projections. It will be important to tease apart the cell-type and circuitry-specific mechanisms underlying the effects of SCN-mediated dampening of rhythms on increased anxiety-like behavior. A more comprehensive understanding of the role of circadian rhythms and the SCN in regulating anxiety may lead to novel chronotherapeutics to treat anxiety disorders.

## Data Availability Statement

The original contributions presented in the study are included in the article/Supplementary Material, further inquiries can be directed to the corresponding author/s.

## Ethics Statement

The animal study was reviewed and approved by University of Pittsburgh IACUC.

## Author Contributions

CV, KP, HZ, RL, and CM designed the experiments. CV, KP, AC, MH, DB-K, and LD were involved in data collection. CV, KP, LE, MH, JB, LD, RL, and CM contributed to the data analysis. All authors contributed to the article and approved the submitted version.

## Conflict of Interest

The authors declare that the research was conducted in the absence of any commercial or financial relationships that could be construed as a potential conflict of interest.

## Publisher’s Note

All claims expressed in this article are solely those of the authors and do not necessarily represent those of their affiliated organizations, or those of the publisher, the editors and the reviewers. Any product that may be evaluated in this article, or claim that may be made by its manufacturer, is not guaranteed or endorsed by the publisher.
