# Supplementary material for: The Suprachiasmatic Nucleus Regulates Anxiety-Like Behavior in Mice
Source: Front Neurosci. 2022 Jan 20;15:765850. doi: 10.3389/fnins.2021.765850 (PMC8811036; doi:10.3389/fnins.2021.765850)
Supplement: Supplementary file 1 [file Data_Sheet_1.PDF]

Supplementary Materials

Table 1. Summary of statistics

Main Figure	Statistics
1B	$t_{(9)} = 7.91$, $p < 0.0001$
1F	$t_{(20.76)} = 7.79$, $p < 0.0001$
1G	$t_{(28)} = 4.01$, $p < 0.001$
3D	$t_{(19)} = 2.21$, $p = 0.04$
3E	$t_{(19)} = 1.29$, $p = 0.21$
5A	$t_{(19)} = 5.22$, $p < 0.0001$
5D	$t_{(19)} = 3.55$, $p < 0.01$
5G	$t_{(19)} = 4.57$, $p < 0.001$
6B	$t_{(8)} = 0.72$, $p = 0.49$
6C	$t_{(8)} = 0.49$, $p = 0.64$
6D	$t_{(10)} = 2.25$, $p < 0.05$
6E	$t_{(10)} = 2.38$, $p < 0.05$

Supplementary Figures	Statistics
2A	$U = 96$, $p = 0.52$
2B	$t_{(23.74)} = 2.11$, $p < 0.05$
3E	genotype x stimulation, $F_{(1,24)} = 39.68$, $p < 0.0001$
3F	genotype x stimulation, $F_{(1,24)} = 4.94$, $p < 0.05$
6A	$U = 48$, $p = 0.65$
6B	$t_{(19)} = 0.86$, $p = 0.40$

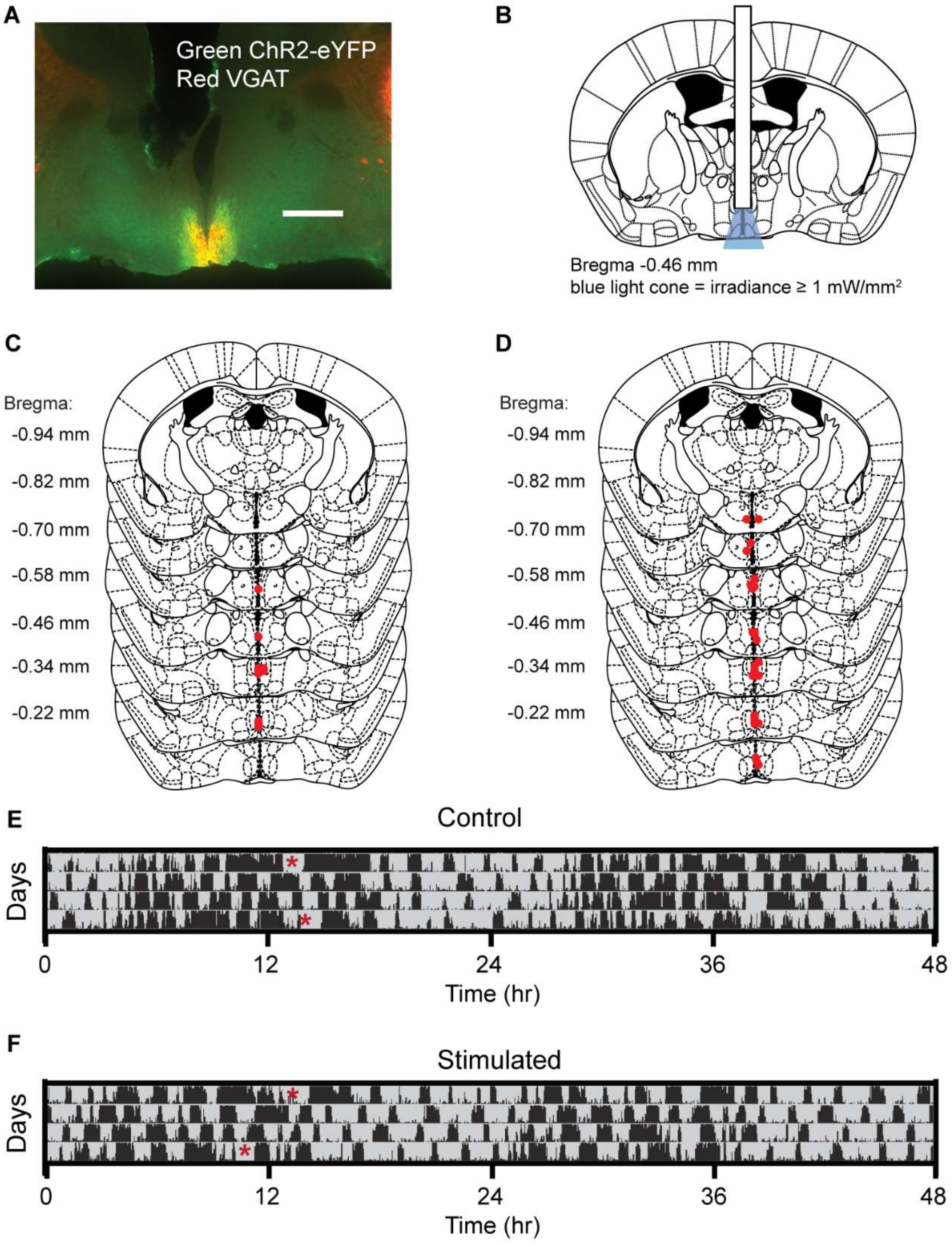
A
Green ChR2-eYFP
Red VGAT
B
Bregma -0.46 mm
blue light cone = irradiance ≥ 1 mW/mm²
C
Bregma:
-0.94 mm
-0.82 mm
-0.70 mm
-0.58 mm
-0.46 mm
-0.34 mm
-0.22 mm
D
Bregma:
-0.94 mm
-0.82 mm
-0.70 mm
-0.58 mm
-0.46 mm
-0.34 mm
-0.22 mm
E
Control
Days
0
12
24
36
48
Time (hr)
F
Stimulated
Days
0
12
24
36
48
Time (hr)

Supplementary Figure 1. **Localization of ChR2 in the SCN, blue light spread and placements for mice stimulated at CT21. (A)** Image showing the localization of VGAT and ChR2-eYFP in a coronal brain slice containing the SCN from a *Vgat*-Cre;ChR2 mouse implanted with an optic fiber. Scale bar = 500 μm. **(B)** Illustration of a coronal brain slice containing the SCN showing the ML and DV spread of blue light with an irradiance ≥ 1 mW/mm^2 emitting from the fiber tip which is sufficient to activate ChR2 (Paxinos and Franklin, 2001). **(C)** Red points on the coronal brain slices indicate the fiber placements above the SCN of mice that received acute SCN optogenetic stimulation at CT21 for assessment of c-Fos-positive cells. (**D)** Red points on the coronal brain slices indicate the fiber placements above the SCN of mice that received chronic SCN optogenetic stimulation at CT21. **(E)** Actogram displaying only homecage activity between sham stimulations 2 and 3 of a control *Vgat*-Cre;ChR2 mouse. **(F)** Actogram displaying only homecage activity between stimulations 2 and 3 of a *Vgat*-Cre;ChR2 mouse.

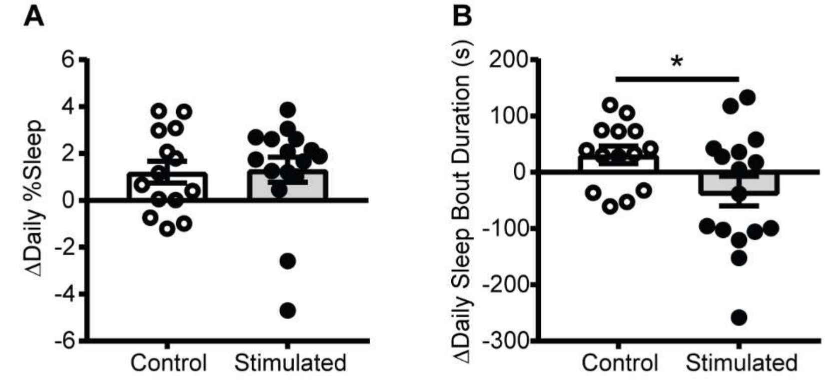

Supplementary Figure 2. Chronic SCN optogenetic stimulation at CT21 decreased sleep bout duration, but not total sleep time relative to sham-stimulated controls. (A) Change in sleep parameters were measured relative to baseline in DD. Control and stimulated mice exhibited similar increases in average daily % time spent sleeping. **(B)** Stimulated mice showed a decrease in the change in average daily sleep bout duration relative to control mice n = 14-16 *Vgat*-Cre;ChR2 mice. *$p < 0.05$.

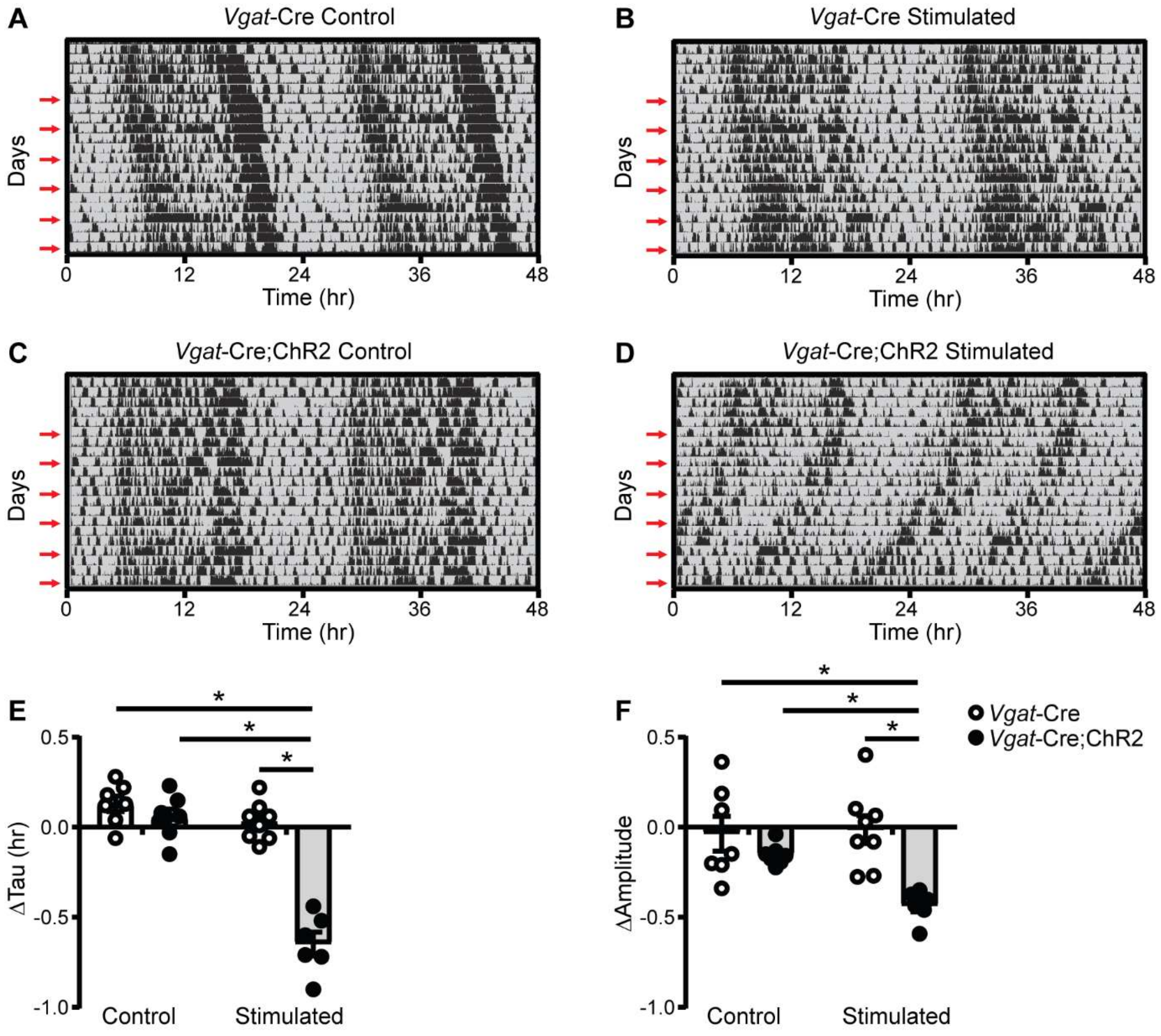

Supplementary Figure 3. Optogenetic stimulation of the SCN at CT21 decreased the period and dampened the amplitude of homecage activity rhythms in *Vgat*-Cre;ChR2 mice relative to *Vgat*-Cre-stimulated and sham-stimulated controls. Representative double-plotted actograms of homecage activity of a **(A)** *Vgat*-Cre homozygous sham control, **(B)** *Vgat*-Cre homozygous stimulated, **(C)** *Vgat*-Cre;ChR2 heterozygous sham control and **(D)** *Vgat*-Cre;ChR2 heterozygous stimulated mouse. Gray shading indicates when lights were off and red arrows indicate the days when stimulations or sham stimulations occurred at CT21. **(E-F)** Change in homecage activity circadian parameters were measured relative to baseline in DD. Only *Vgat*-Cre;ChR2 mice that received blue light pulses onto the SCN showed a significant decrease in the change in homecage activity tau and amplitude relative to controls. n = 6-8 *Vgat*-Cre homozygous or *Vgat*-Cre;ChR2 heterozygous mice. *$p < 0.05$ by Tukey's test.

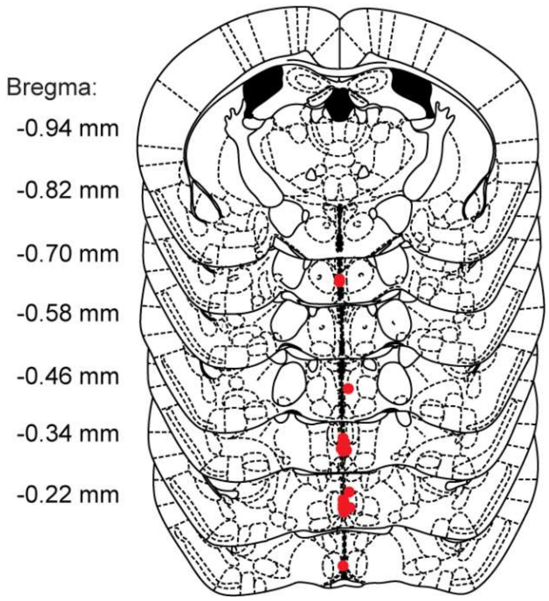

Supplementary Figure 4. Optic fiber placements for the control experiment to determine the effects of chronic optogenetic stimulation of the SCN at CT21 on the homecage activity rhythms of *Vgat*-Cre homozygous and *Vgat*-Cre;ChR2 heterozygous mice.

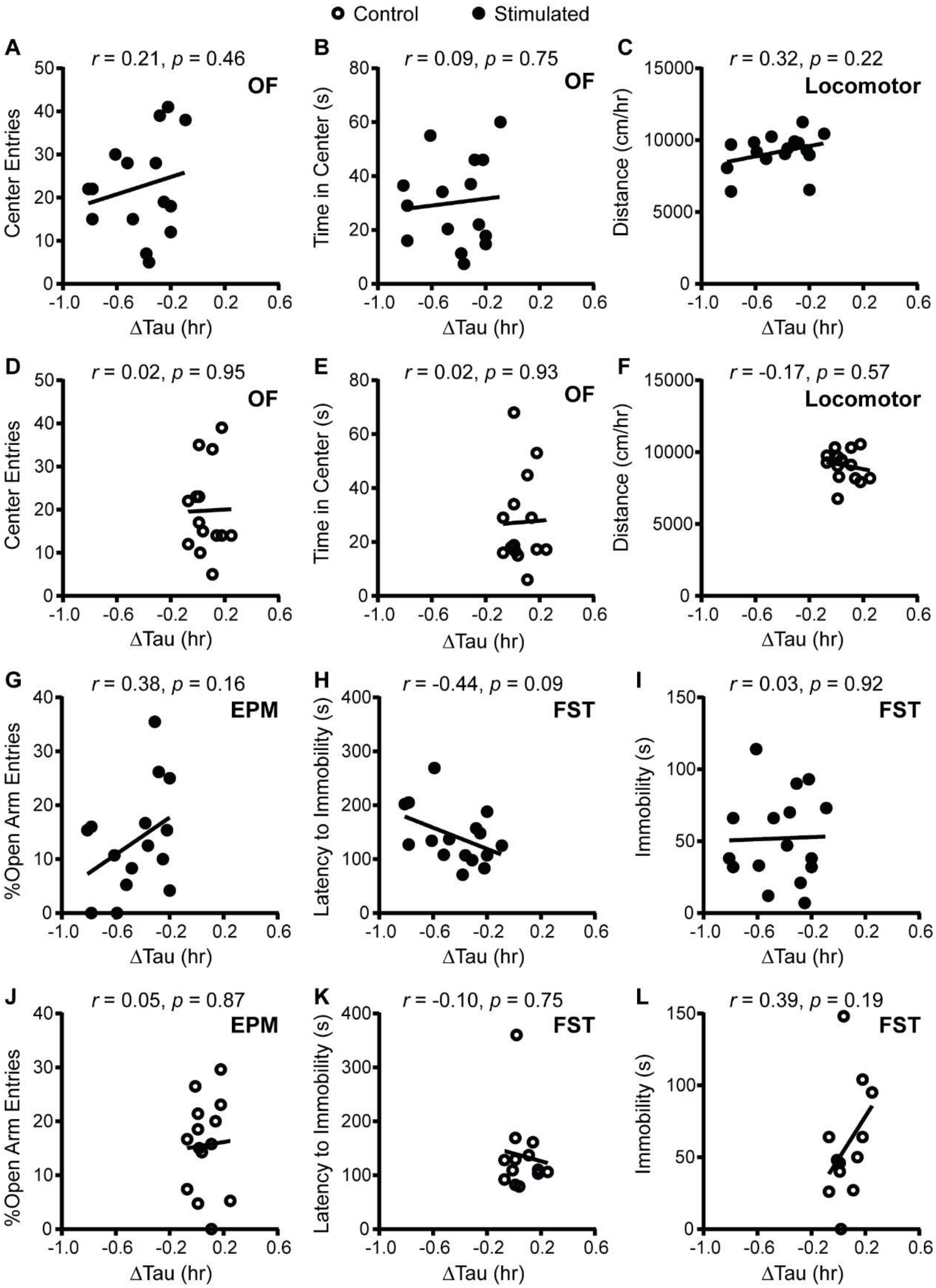

Supplementary Figure 5. No correlations were observed between the change in homecage activity tau and behavior in mice that received stimulations or sham stimulations of the SCN at CT21. (A-B, D-E) open field, **(C, F)** locomotor, **(G, J)** elevated plus maze, **(H-I, K-L)** forced swim test. *n* = 13-16 *Vgat*-Cre;ChR2 mice.

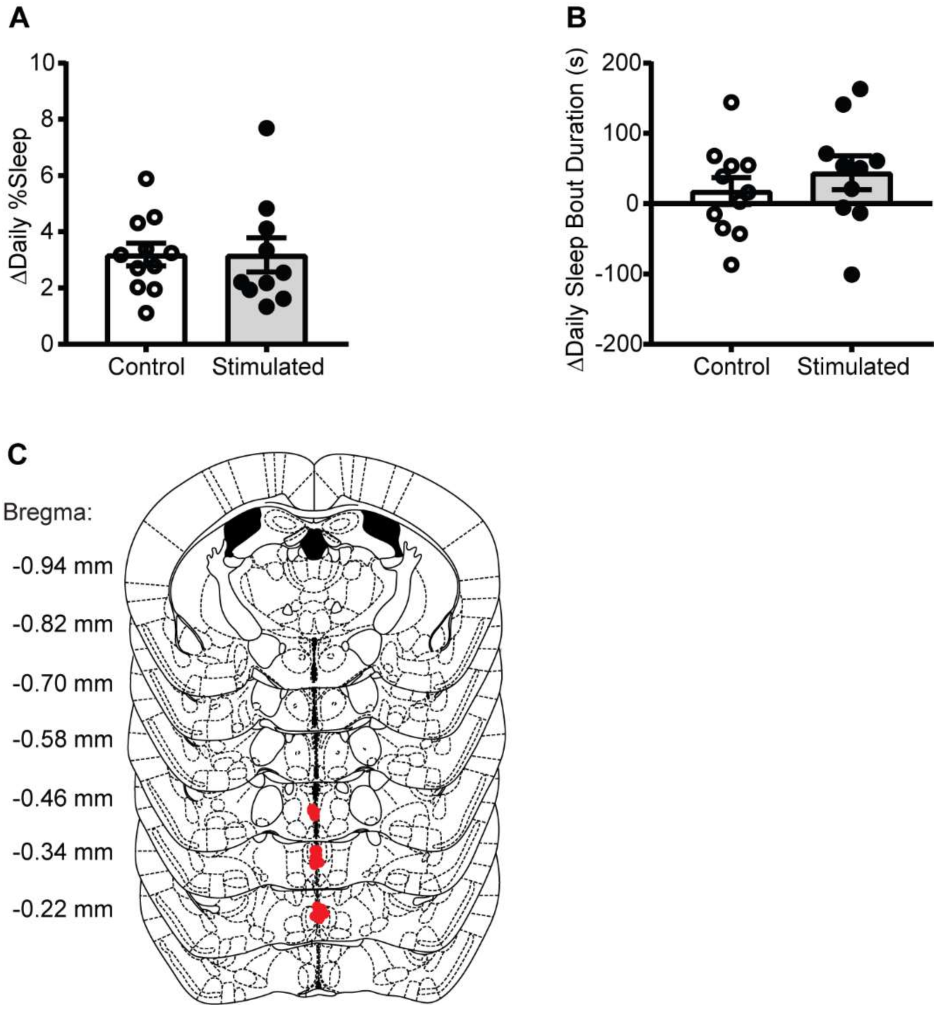

Supplementary Figure 6. Chronic stimulation of the SCN at unpredictable times during the dark phase did not affect measures of sleep relative to controls. (A) Change in sleep parameters were measured relative to baseline in LD. Control and stimulated mice exhibited similar increases in average daily % time spent sleeping. **(B)** Control and stimulated mice also showed similar changes in average daily sleep bout duration. **(C)** Red points on the coronal brain slices indicate the fiber placements in the unpredictable dark phase SCN optogenetic stimulation experiments. *n* = 10-11 *Vgat*-Cre;ChR2 mice.

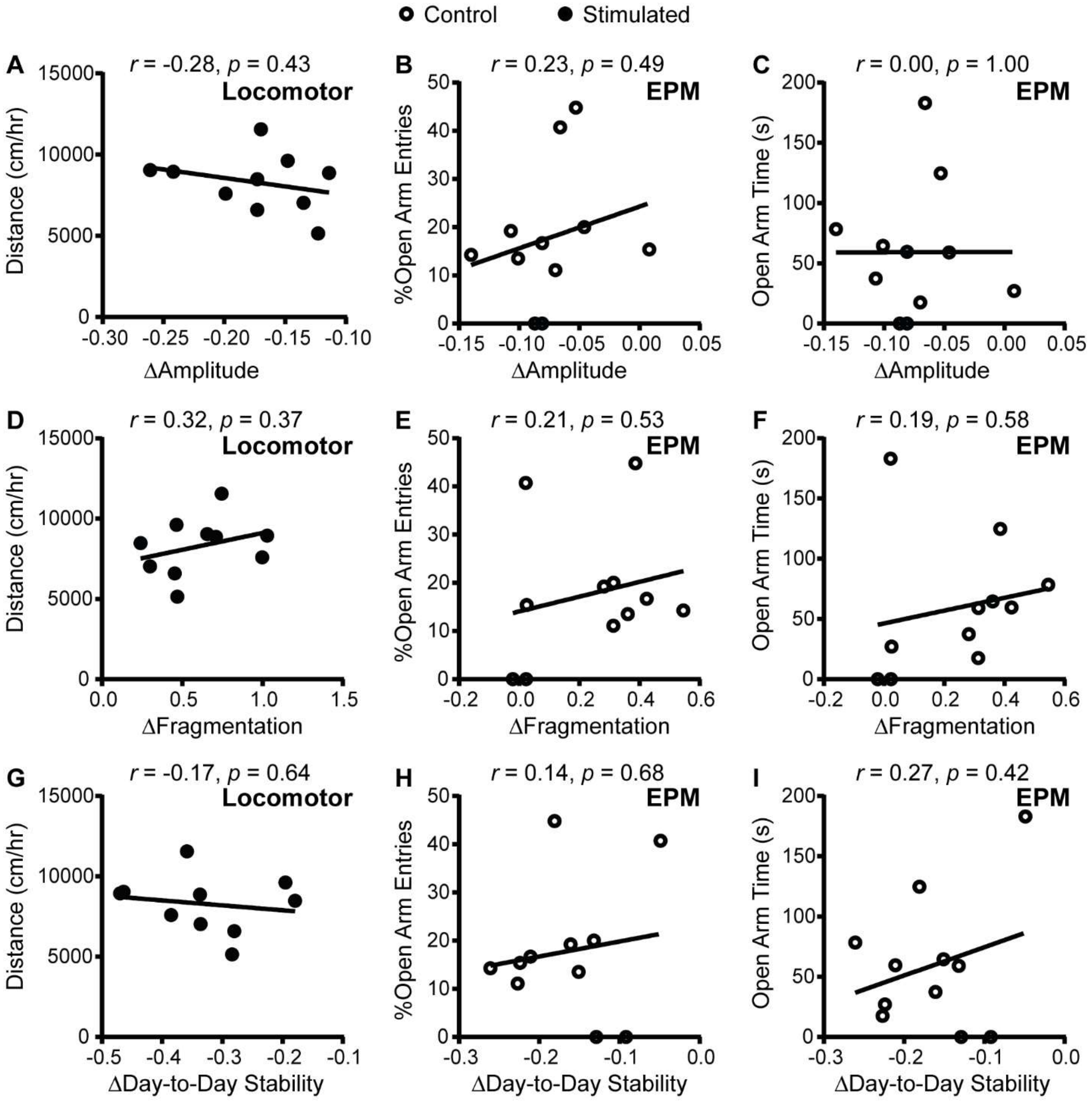

Supplementary Figure 7. Correlations between non-parametric analysis measures of homecage activity rhythms and behavior. (A) Correlations between changes in homecage activity amplitude and locomotor activity in a novel environment and **(B-C)** behavior in the elevated plus maze. **(D)** Correlations between changes in homecage activity rhythm fragmentation and locomotor activity in a novel environment and **(E-F)** behavior in the elevated plus maze. **(G)** Correlations between changes in homecage activity rhythm day-to-day stability and locomotor activity in a novel environment and **(H-I)** behavior in the elevated plus maze. n = 9-11 *Vgat*-Cre;ChR2 mice.
